# Elevated CO_2_ increases photosynthesis in fluctuating irradiance regardless of photosynthetic induction state

**DOI:** 10.1093/jxb/erx357

**Published:** 2017-10-13

**Authors:** Elias Kaiser, Dianfan Zhou, Ep Heuvelink, Jeremy Harbinson, Alejandro Morales, Leo F M Marcelis

**Affiliations:** 1 Horticulture and Product Physiology, Department of Plant Sciences, Wageningen University, AA Wageningen, The Netherlands; 2 Centre for Crop Systems Analysis, Department of Plant Sciences, Wageningen University, AK Wageningen, The Netherlands

**Keywords:** Carbon dioxide, climate change, dynamic photosynthesis, fluctuating light, photosynthetic induction, sine wave, tomato

## Abstract

Leaves are often exposed to fluctuating irradiance, which limits assimilation. Elevated CO_2_ enhances dynamic photosynthesis (i.e. photosynthesis in fluctuating irradiance) beyond its effects on steady-state photosynthesis rates. Studying the role of CO_2_ in dynamic photosynthesis is important for understanding plant responses to changing atmospheric CO_2_ partial pressures. The rise of photosynthesis after a step-wise increase to 1000 μmol m^–2^ s^–1^, the loss of photosynthetic induction after irradiance decreases, and rates of photosynthesis during sinusoidal changes in irradiance were studied in tomato (*Solanum lycopersicum* L.) leaves, using three CO_2_ partial pressures (200, 400, and 800 µbar). Initial irradiance was set to 0, 50, 100, and 200 μmol m^–2^ s^–1^ to vary the initial induction state. Most responses at 200 µbar were not different from those at 400 µbar. In contrast, CO_2_ at 800 µbar increased the relative carbon gain by 12% after an increase in irradiance, decreased the loss of photosynthetic induction by 14%, and increased dynamic photosynthesis during sine waves by 17%, compared with 400 µbar. These effects were additional to steady-state effects of elevated CO_2_ on photosynthesis. The enhancement of dynamic photosynthesis rates by elevated CO_2_ may therefore additionally increase photosynthesis in a future, CO_2_-enriched climate.

## Teaser

Elevated CO_2_ enhances the rate with which photosynthesis responds to stepwise irradiance increases, regardless of the initial photosynthetic induction state, and slows down induction loss after switches to low irradiance.

## Introduction

Carbon dioxide is the substrate of the carboxylation reaction that results in photosynthetic carbon fixation. It is indispensable for carbohydrate synthesis and, ultimately, growth of cyanobacteria, algae, and plants. Atmospheric CO_2_ (*C*_a_) is rising by ~1–2 µbar year^-1^ (IPCC, 2013), and much effort has been directed towards understanding how plant growth in agricultural and natural ecosystems will respond to elevated *C*_a_ (Long *et al.*, 2004; Ainsworth and Rogers, 2007). The positive effects of an elevated CO_2_ partial pressure on steady-state photosynthesis (*A*) in C_3_ species have long been recognized, and are due to a higher rate of carboxylation, and a reduction of the rate of the competing oxygenation reaction, at Rubisco (Long *et al.*, 2004). Crucially, short- and long-term elevated *C*_a_ enhances *A* in a fluctuating irradiance more strongly than under steady-state conditions (Kaiser *et al.*, 2015; Tomimatsu and Tang, 2016), in plants grown at both ambient and at elevated *C*_a_, which is another beneficial yet frequently overlooked factor when considering climate change.

Irradiance incident on a leaf often fluctuates as the sun, clouds, canopies, or the leaf itself, move (Pearcy, 1990; Smith and Berry, 2013), affecting electron transport, the activity of enzymes in the Calvin cycle and sugar metabolism, as well as stomatal conductance (*g*_s_; Stitt and Grosse, 1988; Pearcy *et al.*, 1996; Kaiser *et al.*, 2015, 2016; Kromdijk *et al.*, 2016). The processes that control *A* react to changes in irradiance with time constants in the order of seconds to minutes and, as a result, *A* does not react instantaneously to sudden changes in irradiance. Instead, its rate of change is determined by the rate of change of different processes limiting *A* at that transient. In a shade-adapted leaf that is exposed to a sudden increase in irradiance, the buildup of ribulose-1,5-bisphosphate (RuBP) in the Calvin cycle, the activation of Rubisco, and stomatal opening typically limit the rate of increase in *A*. They do so during different periods of the time course that *A* increases in high irradiance (Pearcy *et al.*, 1996). How quickly *A* changes in response to changes in irradiance furthermore depends on the induction state of the leaf, which is set by the activity of Calvin cycle enzymes and *g*_s_ (Pearcy *et al.*, 1996). Accordingly, *A* in a shade-adapted leaf responds more quickly to an increase in irradiance than *A* in a dark-adapted leaf (Kaiser *et al.*, 2016). As irradiance decreases exponentially throughout a canopy (e.g. Niinemets, 2007; Sarlikioti *et al.*, 2011), the photosynthetic induction state deep in a canopy will be lower than that in the upper part of the canopy. Also, shade-adapted leaves have the same kinetics of induction of *A* as sun leaves (Urban *et al.*, 2007). Notably, integrated *A* deep in the canopy depends more strongly on irradiance fluctuations than high in the canopy, as average irradiance is lower in lower leaf layers (Pearcy *et al.*, 1990).

Several studies have investigated the effects of *C*_a_ on the dynamic responses of *A* and *g*_s_ to changes in irradiance (Chazdon and Pearcy, 1986; Fukayama *et al.*, 1998; Naumburg and Ellsworth, 2000; Naumburg *et al.*, 2001; Leakey *et al.*, 2002; Košvancová *et al.*, 2009; Holišová *et al.*, 2012; Tomimatsu and Tang, 2012; Marín *et al.*, 2014; Tomimatsu *et al.*, 2014; Kaiser *et al.*, 2016; Soleh *et al.*, 2016). The beneficial effects of elevated *C*_a_ (~700 µbar) on *A* after stepwise changes in irradiance have been estimated to be in the order of 5–7% (Leakey *et al.*, 2002; Tomimatsu *et al.*, 2014); these values are additional to enhancement effects on steady-state *A* due to increased *C*_a_. They were caused by faster photosynthetic induction after increases in irradiance, by higher rates of post-illumination CO_2_ fixation, and a decreased post-illumination CO_2_ burst, after decreases in irradiance (Leakey *et al.*, 2002; Tomimatsu *et al.*, 2014). Also, the loss of photosynthetic induction (e.g. deactivation of enzymes and closure of stomata in low irradiance) during the first 5–12 min after a decrease in irradiance was reduced by elevated *C*_a_ (Naumburg and Ellsworth, 2000; Leakey *et al.*, 2002). Importantly, all of these studies only used stepwise changes between two irradiance levels: a low irradiance (‘background irradiance’) and a high, typically saturating, irradiance (‘inducing irradiance’). However, it may be that the effect of elevated *C*_a_ in fluctuating irradiance is not similar across different photosynthetic induction states, such as what occurs in a canopy. If this were true, then predictions of whole-canopy dynamic *A* would be greatly complicated as *A* at different leaf layers would react at different rates to a change in irradiance.

We used tomato (*Solanum lycopersicum* L.) leaves to study the effects of *C*_a_ on *A* in fluctuating irradiance. We compared photosynthetic responses to stepwise increases and decreases in irradiance, using three CO_2_ partial pressures and four levels of background irradiance. Additionally, we exposed leaves to sinusoidal changes in irradiance of several periods. We hypothesized that increases in either CO_2_ partial pressure or background irradiance would increase the rate with which *A* would increase after a stepwise increase in irradiance, and that an increase in both would reduce the loss of photosynthetic induction after a stepwise decrease in irradiance. Further, we hypothesized that there would be no interaction between the effects of CO_2_ partial pressure and background irradiance on dynamic photosynthesis.

## Materials and methods

### Plant material

Tomato seeds (*Solanum lycopersicum* ‘Cappricia’; Rijk Zwaan, De Lier, The Netherlands) were germinated in Rockwool plugs (Grodan, Roermond, The Netherlands), which after a week were transferred to Rockwool cubes (10 cm×10 cm×7 cm; Grodan). Plants were grown in a climate chamber in 16/8 h (day/night) photoperiod, 22/20 °C (day/night) temperature, 70% relative humidity, and 320 µmol m^–2^ s^–1^ photosynthetically active radiation (PAR), measured at table height. CO_2_ partial pressure during plant growth was not controlled but was 400 µbar on average. Irradiance was provided by a mixture of white, red, and far-red light-emitting diodes (LEDs) with emission peaks at 440, 550, 660, and 735 nm (GreenPower LED production module deep red/white 120; Philips, Eindhoven, The Netherlands). Rockwool cubes were standing in a layer (height: 1–2 cm) of nutrient solution (Yara Benelux BV, Vlaardingen, The Netherlands), which was replenished every 1–2 d and contained 12.4 mM NO_3_^–^, 7.2 mM K^+^, 4.1 mM Ca^2+^, 3.3 mM SO_4_^2–^, 1.8 mM Mg^2+^, 1.2 mM NH_4_^+^, 1.1 mM PO_4_^3–^, 30 μM BO_3_^3–^, 25 μM Fe^3+^, 10 μM Mn^2+^, 5 μM Zn^2+^, 0.75 μM Cu^2+^, and 0.5 μM MoO_4_^2–^ (electrical conductivity 2.1 dS m^–1^, pH 5.5). When plants were between 5 and 6 weeks old, leaves 4 and 5, counting from the bottom, were used for measurements. At this stage, growth of these leaves was almost complete (data not shown).

### Experiments and measurements

Experiments were performed in a laboratory, using the LI-6400 photosynthesis system (Li-Cor Biosciences, Lincoln, NE, USA) equipped with a fluorescence chamber (Li-Cor Part No. 6400-40, area: 2 cm^2^). While all plants were grown at 400 µbar, different *C*_a_ were used in the leaf cuvette at three partial pressures: low (200 µbar), ambient (400 µbar), and elevated C_a_ (800 µbar). Other conditions in the measuring cuvette were: 22 ± 0.2 °C cuvette temperature, 70 ± 3% relative humidity, and flow rate of 500 µmol s^–1^. All data were corrected for leaks of CO_2_ into or out of the cuvette, by using dried leaves (Long and Bernacchi, 2003).

Photosynthetic induction was analysed by using stepwise changes between two irradiances, whereby the inducing irradiance was always 1000 µmol m^–2^ s^–1^. The background irradiance was used as a treatment factor and was applied in four levels: 0, 50, 100, and 200 µmol m^–2^ s^–1^. Irradiance was provided by a mixture of red (90%, peak intensity: 635 nm) and blue LEDs (10%, peak intensity: 465 nm). Leaves were adapted to the background irradiance, and to either 200, 400, or 800 µbar CO_2_, until *g*_s_ was stable (60–120 min). Then, irradiance was increased (at the same *C*_a_ level as before) and gas exchange parameters were recorded every 1–2 s for 60 min. Furthermore, to analyse changes in electron transport, saturating flashes of 7000 µmol m^–2^ s^–1^ intensity and 1 s duration were applied once every minute in the first 10 min of induction, and once every 2 min thereafter. Later, it was found that the parameters of the saturating flashes were inappropriate to yield accurate electron transport data (for more information, see Loriaux *et al.*, 2013), and these data were therefore omitted from further analysis. The regular application of saturating flashes had no effects on *A* or *g*_s_ (Supplementary Data 1 at *JXB* online). Loss of photosynthetic induction was analysed by using the same irradiance intensities as for photosynthetic induction. After steady-state *A* and *g*_s_ were reached at 1000 µmol m^–2^ s^–1^, leaves were exposed to a given background irradiance for 0.5, 1, 2, 3, 5, 10, 20, or 60 min. Then, irradiance was returned to 1000 µmol m^–2^ s^–1^ and the ratio of *A* reached 60 s after re-illumination to steady-state *A* at 1000 µmol m^–2^ s^–1^ was used to describe the loss of photosynthetic induction. The loss of photosynthetic induction is measured indirectly when using leaf gas exchange: the deactivation of the system can only be assessed after re-illuminating the leaf at different times in low irradiance and recording the rate of *A* increase in high irradiance. During the course of *A* increase after re-illumination, one time point needs to be chosen that describes the loss of photosynthetic induction as a function of time in low irradiance (Valladares *et al.*, 1997). We chose the relative increase in *A* that was recorded 60 s after re-illumination (RI_60_) to express loss of photosynthetic induction, because the rate of *A* 60 s after re-illumination has most often been used in similar studies (Valladares *et al.*, 1997; Zipperlen and Press, 1997; Leakey *et al.*, 2002). For each replicate, the durations of background irradiance were randomized, with the exception of the 60 min period, which was applied at the end of the measurement sequence.

To test the dynamic behaviour of *A* in response to changes in irradiance, leaves adapted to 300 µmol m^–2^ s^–1^ were exposed to sine wave oscillations in irradiance between 100 µmol m^–2^ s^–1^ and 500 µmol m^–2^ s^–1^ for 30 min, using three different periods (1, 3, and 5 min).

### Calculations

The photosynthetic induction state (PI, %) was calculated after Chazdon and Pearcy (1986): transient *A* was expressed as a percentage of the final rate in inducing irradiance (*A*_f_), corrected for *A* in darkness (*A*_d_):

$$PI=\frac{A-A_{d}}{A_{f}-A_{d}}\times 100$$ (1)

The relative increase in *A* (RI, %) after a stepwise increase in irradiance was calculated as:

$$RI=\;\frac{A-A_{i}}{A_{f}-A_{i}}\times 100$$ (2)

where *A*_i_ is the initial steady-state *A* in background irradiance. The index RI makes it possible to compare the rate of change of *A* of leaves with different initial photosynthetic induction states. In leaves that had previously been in darkness undergoing photosynthetic induction, RI and PI are the same (*A*_d_=*A*_i_), but in low irradiance-adapted leaves PI is larger than RI (*A*_d_<*A*_i_). RI was used to describe the rate of *A* increase during induction and the loss of photosynthetic induction of leaves when irradiance was reduced to various background irradiance levels. This loss was measured 60 s after the leaves were re-exposed to inducing irradiance (RI_60_). A sigmoidal function (Zipperlen and Press, 1997) was fitted to the time courses of induction and loss of induction, and to *g*_s_, for each replicate:

$$x_{\left(t\right)}=\frac{x_{min}-x_{max}}{1+{\left(\frac{t}{i}\right)}^{s}}+x_{max}$$ (3)

where $x_{\left(t\right)}$ is the value of RI, RI_60_, or *g*_s_ at time *t* (min), min and max are the asymptotic minimum and maximum of RI, RI_60_, or *g*_s_, respectively; *i* is the inflection point, and *s* is a shape parameter. The best fit of the model was determined by minimizing the root mean squared error (RMSE) of the residuals between model and data, calculated as:

$$RMSE=\;\sqrt{\frac{1}{n}\overset{n}{\underset{i=1}{\sum }}{\left(x_{\left(t\right)}-{\hat{x}}_{\left(t\right)}\right)}^{2}}$$ (4)

where *n* is the number of observed values, *x*_(*t*)_ is the observed value at time *t*, and ${\hat{x}}_{\left(t\right)}\;$ is the predicted value based on the sigmoidal function at time *t*. The sigmoidal function reproduced changes in RI well, with an average RMSE of 1.9% (see Supplementary Table S3). The index RI_60_ was reproduced slightly less well than RI, with an RMSE of 3.6%. The estimated parameters for each replicate were used to determine the time to reach 50% (*t*_50_) or 90% (*t*_90_) of the total change in RI, and to calculate the increase in *A* at elevated *C*_a_ relative to *A* at ambient *C*_a_ (see below). We used the fitted function rather than the original data to determine these parameters, because the signal-to-noise ratio during gas exchange measurements can have strong, artefactual effects on the calculations. Chloroplast CO_2_ partial pressure, *C*_c_, was calculated as:

$$C_{c}=\;C_{i}-\frac{A}{g_{m}}$$ (5)

where *C*_i_ is substomatal CO_2_ partial pressure and *g*_m_ is mesophyll conductance. Mesophyll conductance was determined previously under identical leaf external CO_2_ partial pressures, genotype, and growth conditions (Kaiser *et al.*, 2017), and the steady-state value under 1000 µmol m^–2^ s^–1^ irradiance was used here. Mesophyll conductance was 0.69 mol m^–2^ s^–1^ at 200 µbar, 0.29 mol m^–2^ s^–1^ at 400 µbar, and 0.12 mol m^–2^ s^–1^ at 800 µbar.

The effect of elevated *C*_a_ relative to ambient *C*_a_ on RI or RI_60_ was calculated as follows. Sigmoidal fits to time courses of single replicates were used. An average value per time point across single replicates was calculated every 15 s. Then, the average response during the first 15 min after a switch to inducing (RI) or background irradiance (RI_60_) was calculated, for each background irradiance (four treatment levels) and for either ambient (RI_avg_amb; 400 µbar) or elevated *C*_a_ (RI_avg_elv; 800 µbar). At each background irradiance level, the effect of elevated over ambient *C*_a_ ($C_{a\_effect}$; %) was calculated as

$$C_{a\_effect}=\left(\frac{RI_{avg}ele}{RI_{avg}amb}-1\right)\times 100$$ (6)

Finally, $C_{a\_effect}$ for RI and RI_60_ was averaged across background irradiance levels, and the SEM was calculated. The effect of elevated *C*_a_ on dynamic *A* during sinusoidal PAR changes was calculated by averaging the response of *A* to PAR for 1, 3, and 5 min periods at each *C*_a_ level. Then, for each sine wave period, the $C_{a\_effect}$ on average *A* at either ambient (*A*_avg_amb) or elevated *C*_a_ (*A*_avg_ele) was calculated as

$$C_{a\_effect}=\left(\frac{A_{avg}ele}{A_{avg}amb}-1\right)\times 100$$ (7)

The value for $C_{a\_effect}$ at 5 min periods was then subtracted from those for 1 min and 3 min periods. This was done because the response at 5 min periods was so slow that *A* was in quasi-steady state, while the response of *A* at 1 min and 3 min periods was more dynamic (see the Results for further discussion). Finally, the average and SEM were calculated from the two values at 1 min and 3 min periods.

The parameters diffusional limitation (*L*_D_; %), biochemical limitation (*L*_B_; %), as well as the apparent time constant of Rubisco activation(τ_R_; Woodrow and Mott, 1989) were calculated essentially as in Kaiser *et al.* (2016), with the following changes: for the determination of τ_R_, instead of using a fixed number of data points for the linear correlation between Δtime and Δln($A_{f}$ –$\;A_{C_{i}}^{*}$), this was varied based on visual observation for every data set. This was necessary as the rate of increase of *A* after a stepwise increase in irradiance was strongly dependent on background irradiance and *C*_a_. Therefore, the length, starting point, and endpoint of the linear part of this relationship varied greatly across experimental conditions. The correlations yielded an average *R*^2^ of 0.97 (see Supplementary Table S2), with the lowest *R*^2^ being 0.93. Details on the calculations of *L*_D_, *L*_B_, and τ_R_ can be found in Supplementary Data 2.

### Statistical analysis

The effects of background irradiance and *C*_a_ on parameters of the sigmoidal function, and on *t*_50_ and *t*_90_, were analysed using two-way ANOVA (Genstat 16th edn., VSN International, Hempstead, UK). Then, single-factor effects were analysed using Fisher’s protected least significant difference tests (Genstat). Single-factor effects on simulated RI and RI_60_ were determined by varying the parameters of the sigmoidal model that were significantly affected by each factor level. Then, 1000 random samples with normal distribution and centred on the mean parameter value, with the SEM of that parameter as the SD of the distribution, were generated. The 2.5th and the 97.5th percentile of those 1000 samples were used to generate the 95% confidence interval around the mean of a given treatment effect.

## Results

### *C*_a_ and background irradiance modulate transient *A* after stepwise changes in irradiance

**Fig. 1.**
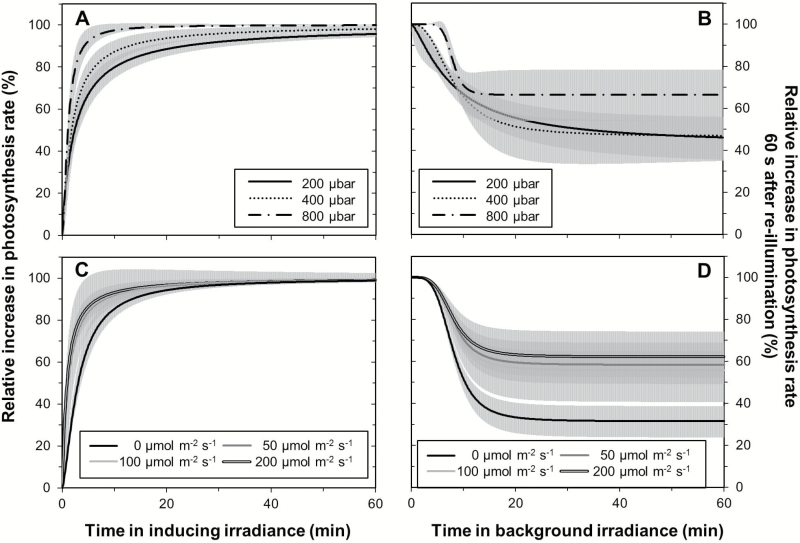
Effects of CO_2_ partial pressure (A, B) and background irradiance (C, D) on the relative increase in *A* after a stepwise increase to inducing irradiance (A, C) and on loss of photosynthetic induction, depicted as the relative increase in *A* 60 s after re-illumination (B, D). Inducing irradiance was 1000 μmol m^–2^ s^–1^ in all cases. In (A) and (B), values are averaged across four background irradiance levels, while in (C) and (D), values are averaged across three CO_2_ partial pressures. In (A) and (C), data were normalized to initial (at 0–200 μmol m^–2^ s^–1^ background irradiance) and final (at 1000 μmol m^–2^ s^–1^) steady-state net photosynthesis rates, while in (B) and (D), data were normalized to the initial steady-state photosynthesis rates (at 1000 μmol m^–^ s^–1^). Curves (averages ±95% confidence intervals) were then fitted to the measured, normalized data using a sigmoidal function (Equation 3). For parameter values of the curve fits, see Supplementary Table S4.

There was no significant interaction either between effects of *C*_a_ and background irradiance on the sigmoidal function fitted to RI and RI_60_ (*P*-values in Table 1), or on *t*_50_ (*P*=0.29) and *t*_90_ (*P*=0.64) of RI. This made it possible to quantify the effects of *C*_a_ and background irradiance separately (Fig. 1). Elevated *C*_a_ (800 µbar) had a stimulating effect on the relative increase in *A* between ~2.5 min and 25 min after a stepwise increase in irradiance (Fig. 1A; Supplementary Fig. S4). Average responses at ambient (400 µbar) and low *C*_a_ (200 µbar) did not differ from each other. *t*_50_ and *t*_90_ decreased with each increase in *C*_a_ (Table 2). *t*_50_ almost halved, while *t*_90_ was only ~27% in elevated compared with low *C*_a_. Between 1.5 min and 4 min after illumination at 1000 µmol m^–2^ s^–1^, dark-adapted leaves showed a significantly slower increase in *A* than low irradiance-adapted leaves (i.e. 50–200 μmol m^–2^ s^–1^), with no difference between the latter (Fig. 1C). In dark-adapted leaves, *t*_50_ was approximately four times larger than in leaves adapted to 200 μmol m^–2^ s^–1^, while *t*_90_ roughly doubled (Table 2).

**Table 1.** Significance of the effects of background irradiance, CO_2_ partial pressure, and their interaction on parameters of sigmoidal fits The sigmoidal function was fitted to data describing the gain and loss of photosynthetic induction (Equation 3).

Irradiance change	Process	Model parameter	Background irradiance	CO_2_ partial pressure	Background irradiance×CO_2_ partial pressure
Step increase	Relative increase in net photosynthesis rate (%)	RI_min_	0.259	0.053	0.940
		RI_max_	0.521	**0.001**	0.478
		*i*	**<0.001**	**<0.001**	0.640
		*s*	**0.005**	**<0.001**	0.233
Step decrease	Relative increase in net photosynthesis rate 60 s after re-illumination (%)	RI_60_min_	0.226	0.198	0.358
		RI_60_max_	**<0.001**	**<0.001**	0.072
		*i*	**0.006**	0.900	0.151
		*s*	0.130	**0.008**	0.284

Numbers denote *P*-values; numbers in bold denote significant effects (*P*<0.05).

**Table 2.** Time (min) to reach 50% (*t*_50_) or 90% (*t*_90_) of final, steady-state *A* after a stepwise increase in irradiance, as affected by CO_2_ partial pressure and background irradiance

Factor	Level	*t*_50_	*t*_90_
CO_2_ partial pressure (µbar)	200	1.91 c	14.7 c
	400	1.62 b	10.0 b
	800	1.02 a	3.9 a
	LSD	*0.27*	*3.1*
Background irradiance (μmol m^–2^ s^–1^)	0	2.72 c	13.1 b
	50	1.03 b	7.6 a
	100	0.89 a,b	7.0 a
	200	0.64 a	8.0 a
	LSD	*0.33*	*3.9*

Different letters denote statistically significant differences (*P*=0.05) within either CO_2_ partial pressure (including all means from background irradiance treatments) or background irradiance treatments (including all means from CO_2_ partial pressures), as determined by Fisher’s protected least significant difference (LSD) tests. LSD values (in italics) are also supplied for comparison

Average loss of photosynthetic induction across background irradiances was slowed by elevated *C*_a_ within 2.5–7.5 min after irradiance dropped, while responses at ambient and low *C*_a_ were similar (Fig. 1B). After this initial period, the induction state tended to decrease less strongly in elevated *C*_a_, but because of the large variability across background irradiance treatments (Supplementary Fig. S5) this was not statistically significant. Average loss of photosynthetic induction across *C*_a_ levels was more severe in leaves exposed to darkness compared with leaves at various shade levels, which did not differ from each other (Fig. 1D).

### Underlying processes during photosynthetic induction

**Fig. 2.**
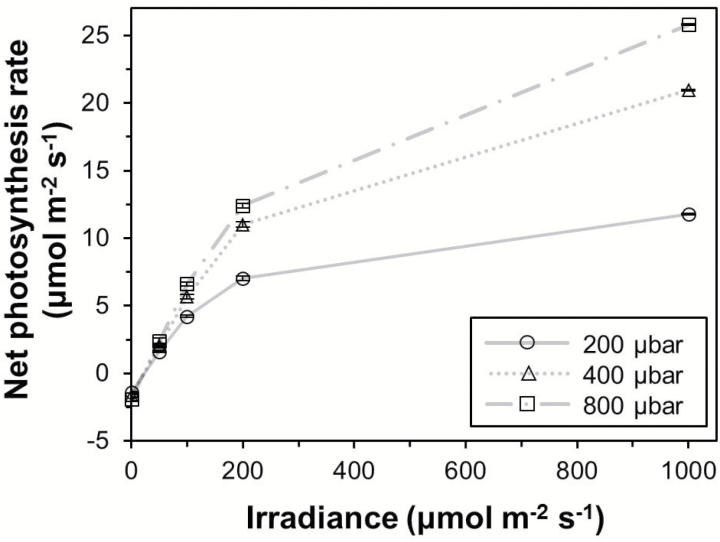
Steady-state response of the net photosynthesis rate to irradiance at three CO_2_ partial pressures. Symbols denote the average ±SEM, *n*=6–28.

**Fig. 3.**
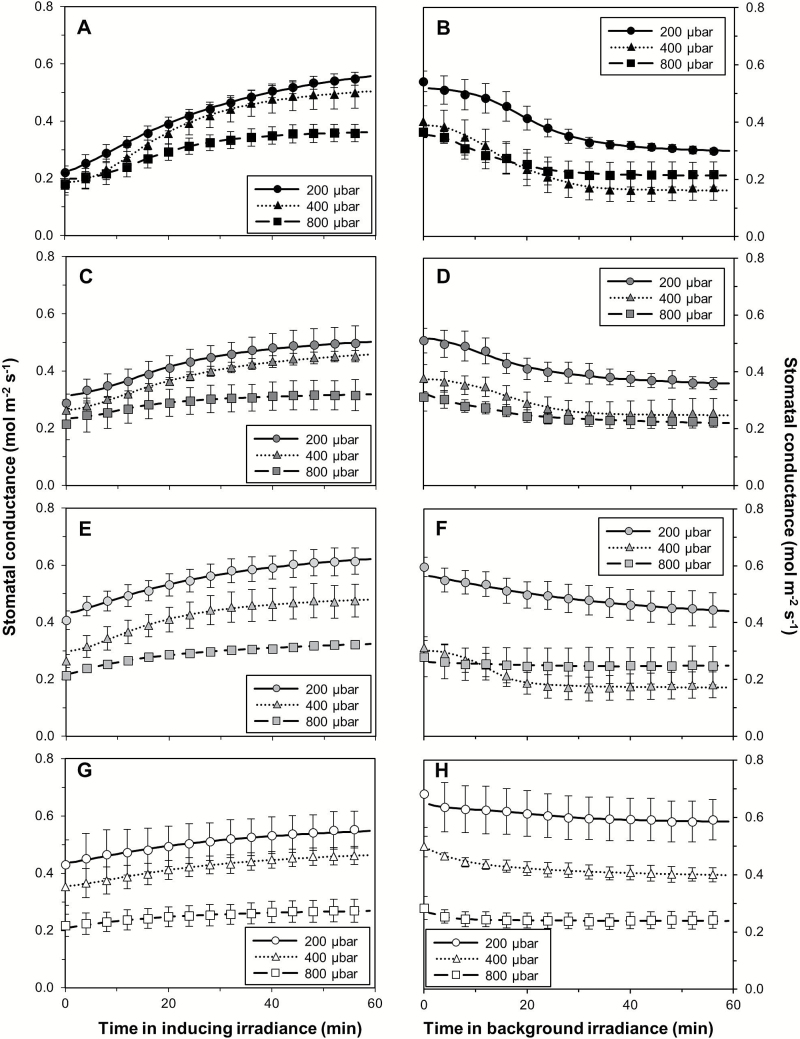
Changes in *g*_s_ after stepwise increases (left panel) and stepwise decreases (right panel) in irradiance at three CO_2_ partial pressures. Background irradiance was 0 (A, B), 50 (C, D), 100 (E,F), or 200 μmol m^–2^ s^–1^ (G, H); inducing irradiance was 1000 μmol m^–2^ s^–1^. Lines denote sigmoidal fits (Equation 3; symbols denote the average ±SEM, *n*=3–5.

As expected, steady-state *A* increased with increases in (background) irradiance, and was further modulated positively by increases in *C*_a_ (Fig. 2). Accordingly, the initial photosynthetic induction state that occurred before a stepwise increase in irradiance was strongly increased by background irradiance (Supplementary Fig. S6). The rate of increase after the switch to inducing irradiance (1000 µmol m^–2^ s^–1^) was, however, increased by both background irradiance and *C*_a_ (Supplementary Fig. S6). The extent of stomatal opening after irradiance increases was larger the greater the difference between inducing and background irradiance was (Fig. 3, left panel). Additionally, low *C*_a_ (200 µbar) led to a more sensitive response of *g*_s_ to irradiance increases, while elevated *C*_a_ (800 µbar) more strongly suppressed differences in stomatal opening.

**Fig. 4.**
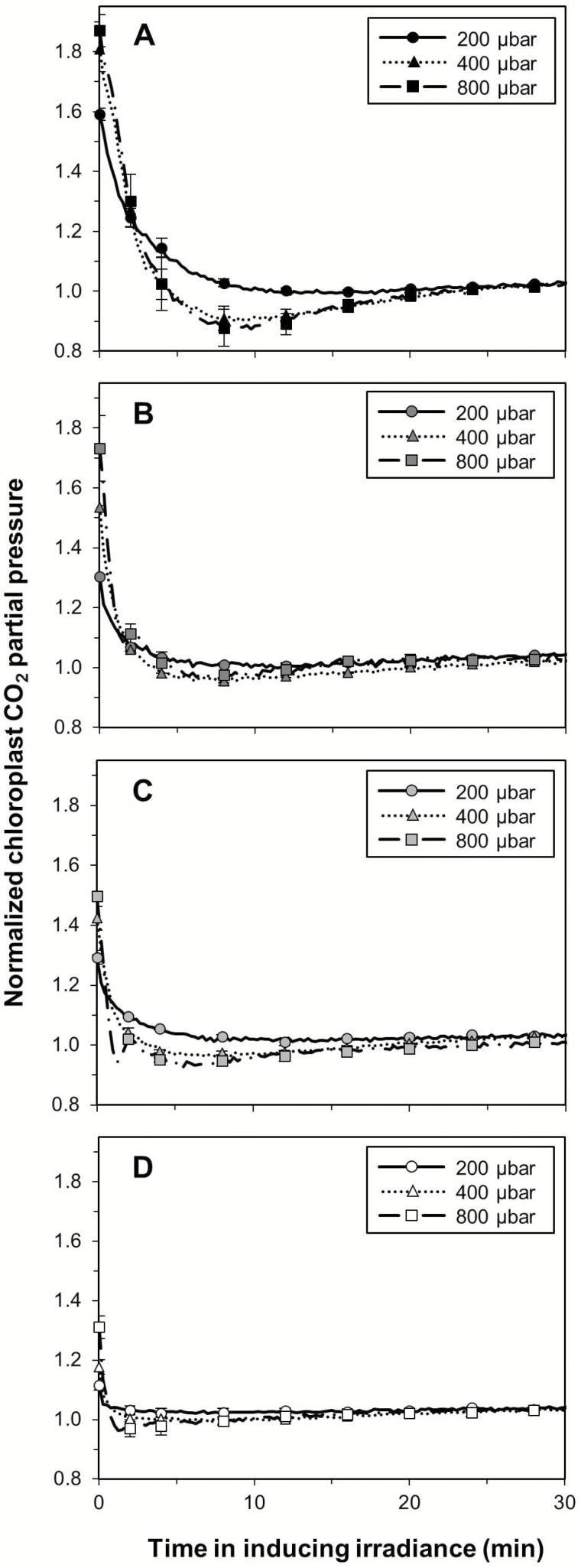
Time courses of chloroplast CO_2_ partial pressure (*C*_c_) after a stepwise increase in irradiance. Background irradiance was 0 (A), 50 (B), 100 (C), or 200 μmol m^–2^ s^–1^ (D); inducing irradiance was 1000 μmol m^–2^ s^–1^. Values were normalized to the final, steady-state *C*_c_. Symbols denote the average ±SEM, *n*=3–5.

**Fig. 5.**
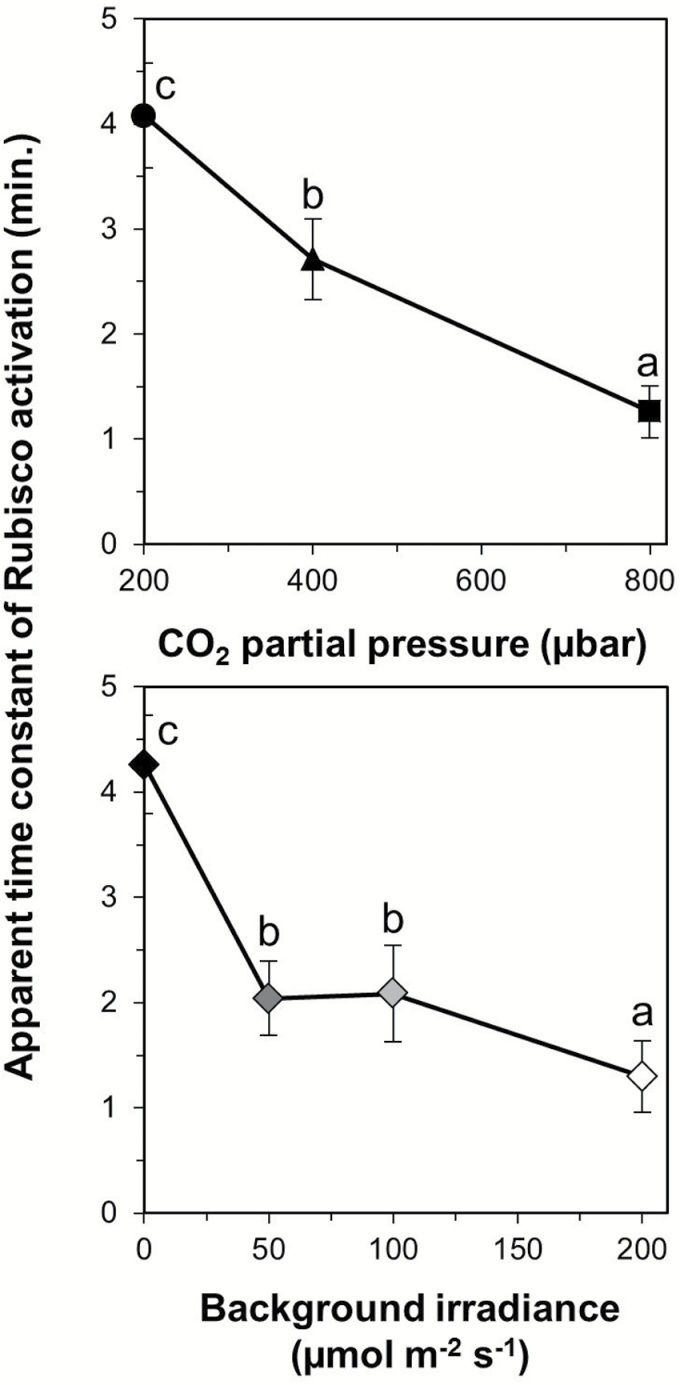
Apparent time constant of Rubisco activation (τ_*R*_) after a stepwise increase in irradiance, as affected by (A) CO_2_ partial pressure and (B) background irradiance. Different letters denote statistically significant (*P*=0.05) differences between treatment levels; symbols denote the average ±SEM, *n*=3–5.

The time courses of *A* and *g*_s_ together affected the normalized chloroplast CO_2_ partial pressure (*C*_c_) after irradiance increases (Fig. 4). Similar to the photosynthetic induction state, the difference between initial and final normalized *C*_c_ decreased with increases in background irradiance; for example, in dark-adapted leaves it changed by ~0.8, while in leaves adapted to 200 µmol m^–2^ s^–1^ this difference was only ~0.2 (Fig. 4A, D). Furthermore, in dark-adapted leaves, *C*_c_ transiently reached levels lower than the final steady-state value during the response to the irradiance increase. These minima were seen ~4–20 min after the irradiance increase in treatments at ambient and elevated *C*_a_, but not at low *C*_a_ (Fig. 4A). Also, these decreases were much less pronounced in low irradiance-adapted leaves at all *C*_a_ levels (Fig. 4B–D). Similar to the changes in *C*_c_, the changes in the indices of diffusional and biochemical limitation, which were derived from transient *A* and *C*_c_, were larger in dark-adapted leaves and decreased as background irradiance increased (Supplementary Fig. S7). The apparent time constant of Rubisco activation [τ_R_; denotes the time to reach 63% of a change in Rubisco activation] showed significant decreases between each *C*_a_ level, reflecting more than three times faster Rubisco activation at elevated compared with low *C*_a_ (Fig. 5A). Generally, τ_R_ also decreased with increases in background irradiance, but showed no significant differences in leaves adapted to 50 µmol m^–2^ s^–1^ and 100 µmol m^–2^ s^–1^ (Fig. 5B).

### Underlying processes during the loss of photosynthetic induction

During the loss of photosynthetic induction, the initial status of the leaf’s photosynthetic capacity was estimated as the induction state that occurred 5 s after re-illumination: this parameter was strongly increased by background irradiance (up to 60% difference between background irradiance levels) and slightly by *C*_a_ (~10% difference due to *C*_a_ levels; Supplementary Fig. S8). This analysis showed that *C*_a_ had a positive impact on the photosynthetic induction level at low irradiance, similar to the response of RI_60_. In the 60 min following a stepwise decrease in irradiance, *g*_s_ generally declined, and this decline increased as the difference between inducing and background irradiance was increased (Fig. 3, right panel).

### Photosynthetic responses to sine waves

**Fig. 6.**
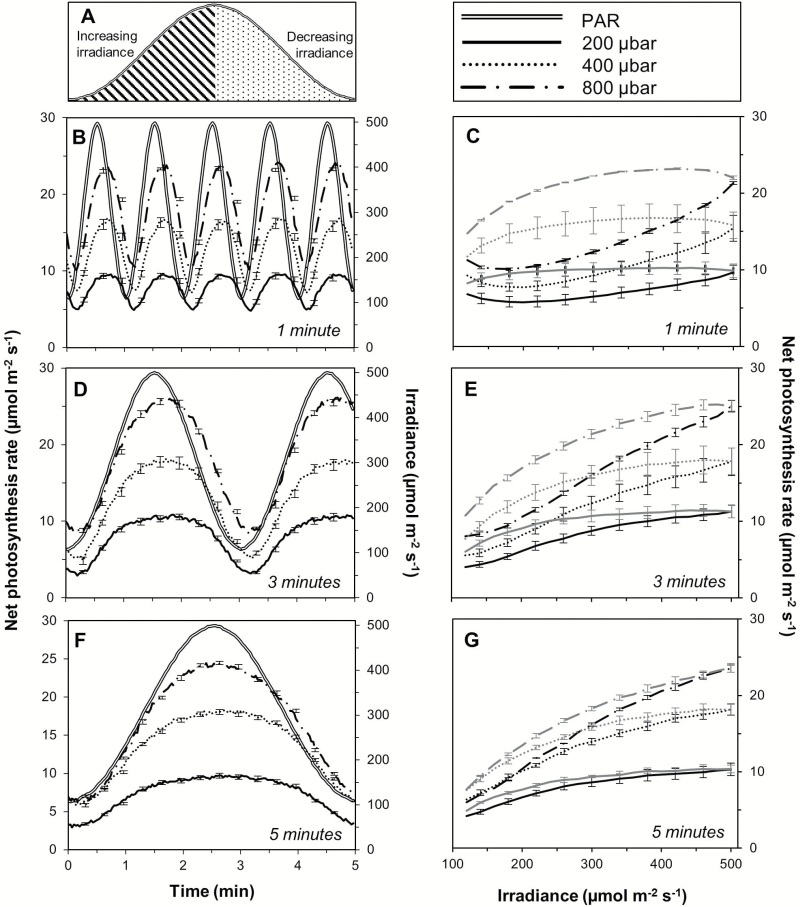
Response of *A* to sinusoidal changes in irradiance, as affected by the period of irradiance changes and CO_2_ partial pressure. (A) Depiction of the concept of half-cycles with the same direction of change in irradiance. Hatched area, half-cycle of increasing irradiance; dotted area, half-cycle of decreasing irradiance. Responses of *A* to 1 min (B, C), 3 min (D, E), and 5 min (F, G) sine waves are shown. *A* during sine waves is plotted as a function of time (B, D, F) and irradiance (C, E, G). In (C), (E), and (G), black lines depict *A* during half-cycles of increasing irradiance while grey lines depict *A* during half-cycles of decreasing irradiance. Error bars depict ±SEM at selected time points or irradiances, *n*=12–15.

Photosynthesis followed the sinusoidal input in irradiance, but with an asymmetric delay that was relatively larger in shorter sine waves compared with longer sine waves (Fig. 6), and differed depending on the direction of irradiance change (see below). Part of this delay was caused by the volume of the measuring system: the system response time for changes in CO_2_ is ~7.5 s (Leakey *et al.*, 2003), and it is presumably unaffected by *C*_a_.

The level of *C*_a_ strongly affected the amplitude (i.e. largest minus smallest value) of *A*, and this was further modulated by the sine wave period. For example, in elevated *C*_a_ the amplitude of *A* was ~14.1 μmol m^–2^ s^–1^ for sine waves with a 1 min period (Fig. 6B), ~17.9 μmol m^–2^ s^–1^ for sine waves with a 3 min period (Fig. 6D), and ~18.3 μmol m^–2^ s^–1^ for sine waves with a 5 min period (Fig. 6F). The relative difference in amplitudes was similar irrespective of sine wave duration: the amplitude of *A* in low *C*_a_ was always ~60% lower than that at elevated *C*_a_, and at ambient *C*_a_ the amplitude of *A* was always ~30% lower than at elevated *C*_a_.

Because of the delay that was revealed by different sine wave frequencies, *A* in the half-cycles during which irradiance decreased was higher than during half-cycles of increasing irradiance. During short sine waves (1 min), *A* was 40–55% higher in half-cycles with decreasing irradiance than in half-cycles with increasing irradiance (Fig. 6C). This difference was smaller for sine waves with 3 min periods (21–26%; Fig. 6E) and even more so for sine waves with 5 min periods (8–11%; Fig. 6G). Additionally, increasing *C*_a_ had a positive effect on the extra carbon gain during half-cycles of decreasing irradiance, such that it enhanced *A* by 3–15%, depending on the sine wave period. It can be deduced from Fig. 6 that during long sine waves (5 min; Fig. 6G), *A* was close to rates expected of a steady-state response to irradiance. In the subsequent analysis of the effects of elevated *C*_a_ on dynamic *A* (see below), the response at 5 min periods was therefore used to account for steady-state effects of elevated *C*_a_.

### Enhancement effects of elevated CO_2_

**Fig. 7.**
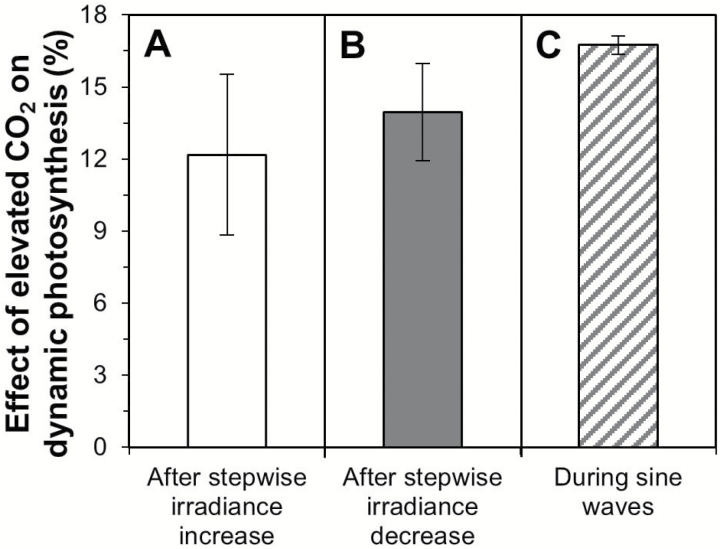
Effects of elevated CO_2_ partial pressure (800 μbar, compared with 400 μbar) on the dynamic response of *A* to changes in irradiance. (A) Effects on the rate of relative increase in photosynthesis rate until 15 min after a stepwise increase in irradiance. (B) Effects on the loss of photosynthetic induction until 15 min after a stepwise decrease to background irradiance. (A) and (B) show averages ±SEM, averaged across background irradiances (*n*=4). (C) Average ±SEM of dynamic responses at 1 min and 3 min periods, relative to dynamic response at the 5 min period (*n*=2).

Relative to ambient *C*_a_, in elevated *C*_a_ the relative increase of *A* was, on average, enhanced by ~12% after increases in irradiance (Fig. 7A). The loss of the photosynthetic induction state after a drop in irradiance was reduced by ~14% in elevated compared with ambient *C*_a_ (Fig. 7B). During sine waves of 1 min and 3 min periods, *A* in elevated *C*_a_ was increased by ~17% compared with ambient *C*_a_ (Fig. 7C; all rates normalized to sine waves of 5 min periods). All these effects of elevated *C*_a_ in non-steady state conditions are additional to the effects of elevated *C*_a_ on steady-state *A*.

## Discussion

### Dynamic photosynthesis in a future, CO_2_-enriched climate

Towards the end of this century, the planet faces an increase in *C*_a_ of 150–450 µbar, depending on the mitigation scenario used (IPCC, 2013). In nature, *A* often operates under fluctuating irradiance and, therefore, its responses to elevated *C*_a_ are highly relevant in a context where irradiance is unstable. In fact, it could be argued that *A* in natural conditions is less likely to be in the steady state than in a non-steady state, and that the dynamic responses of *A* are more relevant to understanding *A* in the field than are steady-state responses. This aspect of non-steady-state photosynthesis is often overlooked, as measurements of the kinetics of change of *A* are less commonly reported and more complex to measure, standardize, and analyse than measurements of steady-state responses of *A*. Our study shows, for the first time, that regardless of its initial photosynthetic induction state, dynamic *A* is increased by 12–17% in elevated (800 μbar) compared with ambient *C*_a_ (400 μbar; Fig 7). This increase is additional to the enhancement that *C*_a_ confers to *A* in the steady state. In canopies, strong gradients in background irradiance occur with canopy depth, and various leaf layers in the canopy are therefore differentially induced before exposure to a sunfleck. Our results indicate that in elevated *C*_a_, the relative rate of *A* increase during a sunfleck will be equal throughout the canopy.

Importantly, this experiment was conducted using plants grown at 400 µbar CO_2_, and thus ignores the potential effects of acclimation to different *C*_a_ that could additionally affect dynamic *A* responses to fluctuating irradiance. For example, acclimation to elevated *C*_a_ often decreases the concentrations of Rubisco and Rubisco activase (Ludewig and Sonnewald, 2000; Chen *et al.*, 2005). The capacity of electron transport also decreases in elevated *C*_a_, but carboxylation is twice as sensitive to a decrease in Rubisco content than it is to a decrease in electron transport (Ainsworth and Rogers, 2007). The increase of *A* during photosynthetic induction may thus be limited by Rubisco activation earlier in the process and remain so for longer in leaves acclimated to high *C*_a_, reducing integrated *A* compared with leaves acclimated to lower *C*_a_. However, this remains to be tested experimentally.

### Larger enhancement effect of elevated CO_2_

Two studies have previously found that elevated *C*_a_ (increasing from 350 µbar to 700 µbar) increased dynamic *A* by 5–7% (Leakey *et al.*, 2002; Tomimatsu *et al.*, 2014), which is roughly half of the effect size observed in the present study (12–17%; Fig. 7). It may be that this difference is caused by the use of species with very different photosynthetic capacity: in both studies, rain forest understorey species were used whose *A* at 350 µbar CO_2_ and an irradiance of 500 µmol m^–2^ s^–1^ was ~4.5 µmol m^–2^ s^–1^ (Leakey *et al.*, 2002; Tomimatsu *et al.*, 2014). The tomato leaves in the present study showed ~4 times larger *A* under similar environmental conditions (~18 µmol m^–2^ s^–1^; Fig. 6G). Thus, leaves in the present study had a larger photosynthetic machinery, implying larger concentrations of Rubisco, Rubisco activase, RuBP regeneration, and electron transport capacity, as well as mesophyll and stomatal conductances. Because of this larger machinery, one could speculate that dynamic *A* in the tomato leaves was able to react more strongly to elevated *C*_a_ than it was in leaves with lower photosynthetic capacity. However, further research is required to confirm this.

### Factors potentially limiting dynamic photosynthesis

The processes that regulate photosynthetic gas exchange react relatively slowly to sudden increases in irradiance (i.e. lightflecks), while *A* drops almost immediately after decreases in irradiance. The combination of those two phenomena causes integrated *A* in fluctuating irradiance to be lower than in constant irradiance. The activity of RuBP regeneration in the Calvin cycle (Sassenrath-Cole and Pearcy, 1992), Rubisco activation state (Yamori *et al.*, 2012; Carmo-Silva and Salvucci, 2013), stomatal conductance (e.g. Kaiser *et al.*, 2016; Li *et al.*, 2016), the relaxation kinetics of photoprotection (Armbruster *et al.*, 2014; Kromdijk *et al.*, 2016), and the rate of sugar synthesis (Stitt and Grosse, 1988) have been shown to be limiting during photosynthetic responses to a changing irradiance, although the specific extent of their limitation depends on the leaf’s photosynthetic induction state (and, hence, its recent irradiance history) and other environmental factors (Kaiser *et al.*, 2017).

An example of how strongly the photosynthetic induction state can modulate a limiting process is the apparent time constant of Rubisco activation (τ_R_), which decreased strongly with increases in background irradiance, and therefore induction state (Fig. 6B). A survey of the literature revealed that this decrease in τ_R_ with increased background irradiance exists for several species using the C_3_ photosynthetic pathway (Supplementary Fig. S9): Arabidopsis (Kaiser *et al.*, 2016), rice (Fukayama *et al.*, 1998), and spinach (Jackson *et al.*, 1991). Altogether, this suggests that the decrease of τ_R_ with an increase in initial photosynthetic induction state is universal, at least in C_3_ species. This may imply that Rubisco is activated faster the smaller the fraction of inactive Rubisco, that an initially larger fraction of active Rubisco activase is beneficial for the activation of the remaining inactive sites of Rubisco, or both. Rubisco was also shown to activate more quickly with increases in *C*_a_ (Fig. 6A), and this has been observed previously (Mott and Woodrow, 1993; Woodrow *et al.*, 1996; Kaiser *et al.*, 2016). This effect may be due to faster carbamylation of Rubisco (Woodrow *et al.*, 1996).

### Leaves in darkness show very different kinetics from leaves in low irradiance

Rates of photosynthesis increase in dark-adapted leaves, and loss of photosynthetic induction in darkness were substantially different from the same processes in various low irradiance levels (Fig. 1; Table 2), which is comparable with responses of soybean leaves, but distinct from those of spinach, *Alocasia macrorrhiza*, and sunflower leaves. In soybean, *A* in dark-adapted leaves exposed to saturating irradiance showed a slower increase than *A* in leaves adapted to a background irradiance of 70–220 µmol m^–2^ s^–1^ (Sassenrath-Cole and Pearcy, 1994), similar to responses in tomato leaves seen in the current study. On the other hand, spinach leaves did not exhibit abrupt changes in induction rates between background irradiances of 0 and ~135 μmol m^–2^ s^–1^ (Jackson *et al.*, 1991). Also, *A*. *macrorrhiza* lost photosynthetic induction much more quickly in darkness than in 10 μmol m^–2^ s^–1^ (Kirschbaum and Pearcy, 1988), and sunflower leaves showed faster increases in photosynthetic induction after adaptation to 10 μmol m^–2^ s^–1^ than to darkness (Kirschbaum *et al.*, 2005). The necessity for a buildup of RuBP pools large enough to support high rates of CO_2_ assimilation (Sassenrath-Cole and Pearcy, 1992, 1994), and a reduced quantum yield of *A* in the first minutes of photosynthetic induction (Kirschbaum *et al.*, 2004, 2005) may be responsible for the observed differences between dark- and low irradiance-adapted leaves. A lower activation state of Rubisco and Rubisco activase in darkness compared with light (see discussion above) may be another reason.

### Conclusions

In conclusion, we show here that elevated CO_2_ partial pressure (800 µbar, compared with 400 µbar) enhances the dynamic response of photosynthesis to a change in irradiance, regardless of initial photosynthetic induction state, and that it does so to a considerable extent (by 12–17%). This effect is additional to the positive effect of elevated CO_2_ partial pressure on steady-state photosynthesis rates. Future increases in ambient CO_2_ partial pressure may therefore benefit carbon assimilation of differently induced leaves in naturally fluctuating irradiance.

## Supplementary data

Supplementary data are available at *JXB* online.

Table S1. Effects of saturating flashes on gas exchange rates during photosynthetic induction.

Table S2. Details of calculations of diffusional limitation, biochemical limitation, and the apparent time constant of Rubisco activation.

Table S3. Goodness of fit of sigmoidal function used on dynamic photosynthesis data.

Table S4. Parameters used for sigmoidal fits in Figs 1–3.

Fig. S1. Data used for determination of the parameters *VCmax*, *J*, *TPU* and Γ*.

Fig. S2. Data used for the determination of day respiration.

Fig. S3. Examples of determination of the apparent time constant of Rubisco activation (*τR*), in four induction curves (at 400 μbar CO2), as affected by background irradiance.

Fig. S4. Photosynthetic induction state after stepwise increases in irradiance.

Fig. S5. Diffusional and biochemical limitations after stepwise increases in irradiance.

Fig. S6. Photosynthetic induction state during loss of photosynthetic induction.

Fig. S7. Photosynthesis rates during increasing or decreasing sinusoidal changes in irradiance, plotted against irradiance.

Fig. S8. Relationship between the apparent time constant of Rubisco and background irradiance in several species.

## Conflict of interest

None declared

## Supplementary Material

Supplementary Tables S1-S4 Figures S1-S9

## Abbreviations:

A: net photosynthesis rate
*C*_a_: atmospheric CO_2_ partial pressure
*C*_c_: chloroplast CO_2_ partial pressure
*g*_s_: stomatal conductance
*L*_B_: transient biochemical limitation
*L*_D_: transient diffusional limitation
RI: relative increase in net photosynthesis rates after a stepwise irradiance increase
RI_60_: relative increase in net photosynthesis rates, 60 s after a stepwise irradiance increase
*t*_50_ (*t*_90_): times to reach 50% (90%) of the total change in RI
τ_R_: the apparent time constant of Rubisco activation.
