# Supplementary material for: Elevated CO2 increases photosynthesis in fluctuating irradiance regardless of photosynthetic induction state
Source: J Exp Bot. 2017 Oct 13;68(20):5629–40. doi: 10.1093/jxb/erx357 (PMC5853276; doi:10.1093/jxb/erx357)
Supplement: Supplementary Tables S1-S4 Figures S1-S9 [file erx357_suppl_supplementary_tables_s1-s4_figures_s1-s9.pdf]

Supplementary Data

Effects of saturating flashes on gas exchange rates during photosynthetic induction

To exclude the possibility that saturating flashes affected the rate of photosynthetic induction or rates of stomatal opening, parameters from gas exchange responses (after a 0→1000 µmol m^{-2} s^{-1} stepwise increase) with and without the regular application of saturating flashes were compared, across three CO_2 partial pressures (Table S1). Only two parameters were significantly different between data sets: initial g_s in darkness in 400 µbar (which was unaffected by saturating flashes, since they were applied after dark adaptation), and final g_s in light in 200 µbar, where g_s was 0.1 mol m^{-2} s^{-1} lower in the data set where saturating flashes had been applied. All other parameters being the same, this difference seemed small enough to carry on with the analysis of gas exchange data.

Table S1. Effects of application of saturating flashes on parameters of photosynthetic induction and stomatal conductance, average ± SEM (n = 5). Parameters were derived from gas exchange measurements on dark-adapted leaves after 0→1000 µmol m^{-2} s^{-1} stepwise increases. Parameters from induction without flashes ("No Flashes") have been derived from an earlier publication (Kaiser *et al.*, 2017), while parameters derived from induction with periodic (every 1-2 minutes during 60 minutes) application of saturating flashes ("Flashes") are those of the current study. Stars (* = P<0.05) denote statistically significant differences between Flashes and No Flashes, absence of stars denotes lack of significant difference. Abbreviations: IS_{60}; induction state (%) 60 seconds after illumination, t_{A50} and t_{A90}; time (minutes) to reach 50 and 90% of full photosynthetic induction, respectively, t_{gs50} and t_{gs90}; time (minutes) to reach 50 and 90% of final stomatal conductance, respectively, A_i and A_f, steady-state photosynthesis rate (µmol m^{-2} s^{-1}) in darkness and in 1000 µmol m^{-2} s^{-1}, respectively, g_{si} and g_{sf}; steady-state stomatal conductance in darkness and in 1000 µmol m^{-2} s^{-1}, respectively, τ_R; apparent time constant of Rubisco activation (minutes).

	CO_2 partial pressure																				
	200 (µbar)							400 (µbar)							800 (µbar)						
	No Flashes			Flashes				No Flashes			Flashes				No Flashes			Flashes			
IS_{60}	25.7	±	1.4	22.6	±	1.9	ns	21.6	±	1.2	21.2	±	1.0	ns	21.9	±	1.9	27.5	±	2.7	ns
t_{A50}	3.2	±	0.3	3.4	±	0.3	ns	2.6	±	0.1	2.6	±	0.2	ns	2.2	±	0.1	1.8	±	0.1	ns
t_{A90}	18.5	±	1.8	18.7	±	1.3	ns	10.8	±	0.6	13.7	±	1.6	ns	6.2	±	0.1	5.9	±	0.3	ns
t_{gs50}	19.8	±	0.5	19.0	±	1.3	ns	18.7	±	1.4	17.5	±	1.0	ns	18.2	±	1.0	15.8	±	0.5	ns
t_{gs90}	46.7	±	0.6	45.0	±	1.9	ns	38.2	±	2.5	36.7	±	2.2	ns	39.9	±	2.1	34.8	±	1.9	ns
An_d	-1.1	±	0.2	-1.6	±	0.2	ns	-1.6	±	0.1	-1.2	±	0.2	ns	-1.3	±	0.3	-1.3	±	0.2	ns
An_L	11.7	±	0.6	12.2	±	0.4	ns	22.2	±	0.6	22.0	±	0.4	ns	27.1	±	1.0	25.5	±	0.9	ns
gs_d	0.2	±	0.0	0.2	±	0.0	ns	0.3	±	0.0	0.2	±	0.0	*	0.2	±	0.0	0.2	±	0.0	ns
gs_L	0.7	±	0.0	0.6	±	0.0	*	0.6	±	0.0	0.5	±	0.1	ns	0.5	±	0.0	0.4	±	0.0	ns
τ_R	5.1	±	0.7	6.1	±	0.6	ns	4.1	±	0.2	3.8	±	0.3	ns	2.7	±	0.1	2.2	±	0.2	ns

Calculating LD, LB and τ_R

To calculate several parameters, gas exchange data were corrected for transient changes in chloroplast CO_2 partial pressure (C_c). For diffusional limitation (L_D; %), *A* was multiplied by the percentage by which *A* would increase if C_c during induction was equal to C_a ($A^*_{C_a}$), i.e. assuming an infinite conductance to CO_2 diffusion through the leaf (see below). For biochemical limitation (L_B; %) and the apparent rate constant of Rubisco activation (τ_R; min), *A* was multiplied by the percentage by which *A* would increase if transient C_c was similar to final, steady-state C_c ($A^*_{C_c}$), following Woodrow & Mott (1989). Unlike Woodrow and Mott (1989), we used A/C_c relationships instead of A/C_i relationships. The mesophyll conductance (g_m) used to calculate C_c from C_i and *A* during photosynthetic induction was assumed to be specific for the treatment CO_2 partial pressure; this was 0.69 mol m^{-2} s^{-1} at 200 µbar, 0.29 mol m^{-2} s^{-1} at 400 µbar and 0.12 mol m^{-2} s^{-1} at 800 µbar. These values had previously been determined using the variable *J* method (Harley *et al.*, 1992; Kaiser *et al.*, 2016). Also, unlike Woodrow and Mott (1989), for calculations of $A^*_{C_a}$ and $A^*_{C_c}$ no linear relationship between C_c and the CO_2 compensation point (Γ^*, Pa) was assumed. Instead, information from complete A/C_c curves was used to correct *A* using the steady-state, curvilinear response of *A* to C_c. To calculate $A^*_{C_a}$, *A* was corrected for the minimum of either Rubisco activity-limited *A* (A_c), RuBP-limited *A* (A_j) or triose phosphate utilization-limited *A* (A_t) at C_a (in the numerator) and at C_c (in the denominator):

$$A^*_{C_a} = A \cdot \frac{min\{A_c(C_a), A_j(C_a), A_t(C_a)\}}{min\{A_c(C_c),\ A_j(C_c), A_t(C_c)\}} \quad \text{(S1)}$$

A_c, A_j and A_t were calculated after the FvCB model (Farquhar *et al.*, 1980) modified to account for TPU limitation (Sharkey, 1985). In eqns. 6-8, the calculations for *A* at C_a are shown. To calculate $A^*_{C_c}$, C_a in the numerator was replaced by final, steady-state C_c (not shown here):

$$A_c(C_a) = V_{Cmax}\left(\frac{C_a - \Gamma^*}{C_a + K_c \cdot \left(1 + \frac{O}{K_o}\right)}\right) - R_d \quad \text{(S2)}$$

$$A_j(C_a) = J\left(\frac{C_a - \Gamma^*}{4 \cdot C_a + 8 \cdot \Gamma^*}\right) - R_d \quad \text{(S3)}$$

$$A_t(C_a) = 3 \cdot TPU - R_d \quad \text{(S4)}$$

Where V_{Cmax} (168 µmol m^{-2} s^{-1}) is maximum rate of carboxylation by Rubisco, R_d is mitochondrial respiration (1.54 µmol m^{-2} s^{-1}), O is the chloroplast O_2 partial pressure (210 mbar), K_c (21.4 µbar) and K_o (15.4 mbar) are the Michaelis-Menten constants of Rubisco for CO_2 and for O_2, respectively, J (169 µmol m^{-2} s^{-1}) is the rate of electron transport in the absence of regulation and TPU (9.2 µmol m^{-2} s^{-1}) is the triose phosphate utilization rate. Parameters V_{Cmax}, J and TPU were estimated using the method of Sharkey et al. (2007). The first four points of A/C_c curves at 21 kPa O_2 partial pressure (Fig. S1) were used to estimate V_{Cmax} (initial slope), the next five points to estimate J and the uppermost two points to estimate TPU (three replicates per A/C_c curve were used). For the purpose

of A/C_c curve-fitting, g_m was assumed to be constant at 0.29 mol m^{-2} s^{-1}. R_d and Γ^* were determined after Yin et al. (2009; Fig. S2). All values were corrected for respiration from leaf material lying under the gasket of the gas exchange cuvette (Pons and Welschen, 2002). Parameters K_c and K_o were taken from Sharkey et al. (2007); these have been determined on leaves of tobacco (Bernacchi *et al.*, 2001), which is closely related to tomato. L_D was then determined by analogy to stomatal limitation as in Urban et al. (2007)

$$L_D = \frac{A^*_{C_a} - A}{A_f - A_i} \cdot 100 \qquad \text{(S5)}$$

Biochemical limitation (L_B) was calculated by using $A^*_{C_c}$, that is, A corrected for the relative rates of steady-state A at C_{c_f} in the numerator (instead of C_a) and steady-state A at C_c in the denominator of Eqn. S1. L_B was calculated after Urban et al. (2007)

$$L_B = \frac{A_f - A^*_{C_c}}{A_f - A_i} \cdot 100 \qquad \text{(S6)}$$

The apparent time constant of Rubisco activation (τ_R) was calculated after Woodrow and Mott (1989):

$$\tau_R = \frac{\Delta time}{\Delta \ln(A_f - A^*_{C_i})} \qquad \text{(S7)}$$

where Δtime is the duration used for determination of τ_R. Instead of using a fixed number of data points for the linear correlation between $\Delta time$ and $\Delta \ln(A_f - A^*_{C_i})$, as was done in Woodrow and Mott (1989), this was varied based on visual observation for every dataset (Fig. S3, Table S2). This was necessary as the rate of increase of A after a stepwise increase in irradiance was strongly dependent on background irradiance and CO_2 partial pressure. Therefore, the length, starting point and endpoint of the linear part of this relationship varied greatly across experimental conditions. The correlations yielded an average R^2 of 0.97 (Table S2), with the lowest R^2 being 0.97.

Table S2. Parameters describing the correlations between $\Delta\ln(A_f - A^*_{C_c})$ and $\Delta time$ of eqn. S7 $[\tau_R = \Delta time/\Delta\ln(A_f - A^*_{C_i})]$ after a stepwise increase in irradiance. Average ± SEM (n = 3-5). The start and end, as well as the duration, of the correlation were varied with time (see Fig. S3), to obtain highly linear correlations (signified by R^2).

Background irradiance (μmol m^{-2} s^{-1})	CO_2 partial pressure (μbar)	Start (min)			End (min)			Duration (min)			R^2		
0	200	1.13	±	0.05	3.87	±	0.38	2.74	±	0.34	0.98	±	0.01
0	400	1.19	±	0.04	4.57	±	0.12	3.38	±	0.08	0.98	±	0.00
0	800	1.15	±	0.00	4.45	±	0.00	3.30	±	0.00	0.99	±	0.00
50	200	0.50	±	0.00	4.45	±	0.00	3.95	±	0.00	0.97	±	0.01
50	400	0.50	±	0.00	4.45	±	0.00	3.95	±	0.00	0.98	±	0.00
50	800	0.12	±	0.07	1.23	±	0.07	1.12	±	0.09	0.98	±	0.01
100	200	0.78	±	0.17	4.95	±	0.00	4.17	±	0.17	0.95	±	0.01
100	400	0.10	±	0.00	1.72	±	0.12	1.62	±	0.12	0.99	±	0.00
100	800	0.22	±	0.06	0.97	±	0.02	0.75	±	0.06	0.97	±	0.01
200	200	0.20	±	0.06	2.45	±	1.01	2.25	±	1.04	0.91	±	0.01
200	400	0.03	±	0.02	1.55	±	0.34	1.52	±	0.34	0.98	±	0.00
200	800	0.00	±	0.00	0.83	±	0.17	0.83	±	0.17	0.97	±	0.01

Table S3. Goodness of fit of sigmoidal function, as illustrated by the root mean squared error (RMSE, Eqn. 3). The sigmoidal function was fitted to the index RI (relative increase in net photosynthesis rate) during a period of 60 minutes after a stepwise increase in irradiance, and to the index RI_{60} (relative increase in net photosynthesis rate 60 seconds after re-illumination) as a function of time since the stepwise decrease in irradiance. Displayed are the averages, plus the 1st and 3rd percentile of single-replicate values, across CO_2 partial pressures and background irradiance treatments (n = 38-42)

Irradiance change	Index	Root mean squared error (%)		
		Average	1st percentile	3rd percentile
Stepwise increase	Relative increase in net photosynthesis rate (%)	1.9	1.3	2.4
Stepwise decrease	Relative increase in net photosynthesis rate 60 s after re-illumination (%)	3.6	2.4	4.2

Table S4. Parameters used for sigmoidal fits in Figures 1,S4 and S5. For interpretation of parameters, please refer to Eqn. 3.

Figure	CO_2 partial pressure (µbar)	Background irradiance (µmol m^{-2} s^{-1})	Direction of irradiance change	Shape parameter			
				x_{min}	x_{max}	i	*s*
1A	200	all	up	0.0	100.0	2.4	1.0
	400	all	up	0.0	100.0	1.8	1.1
	800	all	up	0.0	100.0	1.1	1.7
1B	200	all	down	100.0	42.4	7.9	1.3
	400	all	down	100.0	46.6	7.9	2.5
	800	all	down	100.0	66.4	7.9	7.7
1C	all	0	up	0.0	100.0	1.5	1.5
	all	50	up	0.0	100.0	1.1	1.1
	all	100	up	0.0	100.0	1.1	1.1
	all	200	up	0.0	100.0	0.9	1.1
1D	all	0	down	100.0	31.4	8.0	3.9
	all	50	down	100.0	58.3	8.0	3.9
	all	100	down	100.0	55.8	8.0	3.9
	all	200	down	100.0	62.1	8.0	3.9
S4A	200	0	up	3.4	105.5	3.8	1.1
	400	0	up	1.4	103.3	3.3	1.3
	800	0	up	6.4	100.7	2.1	2.1
S4B	200	50	up	4.4	102.1	1.5	1.0
	400	50	up	1.4	101.5	1.1	1.1
	800	50	up	3.5	101.9	0.8	1.2
S4C	200	100	up	6.2	104.4	1.6	0.9
	400	100	up	3.7	103.4	1.1	1.0
	800	100	up	5.7	101.5	0.5	1.5
S4D	200	200	up	6.2	105.2	1.8	0.8
	400	200	up	4.4	103.9	0.7	1.0
	800	200	up	8.1	100.0	0.4	1.6
S5A	200	0	down	108.1	26.3	8.4	1.1
	400	0	down	102.7	37.8	6.7	2.2
	800	0	down	109.6	34.6	11.1	1.7
S5B	200	50	down	100.9	64.7	3.7	2.5
	400	50	down	105.7	61.7	2.7	2.0
	800	50	down	108.2	74.4	4.1	6.1
S5C	200	100	down	107.7	64.2	4.4	0.8
	400	100	down	104.2	59.8	9.1	1.1
	800	100	down	111.0	88.0	8.3	8.8
S5D	200	200	down	107.8	76.9	14.8	1.1
	400	200	down	104.5	80.3	8.8	5.5
	800	200	down	105.6	91.0	8.1	14.4

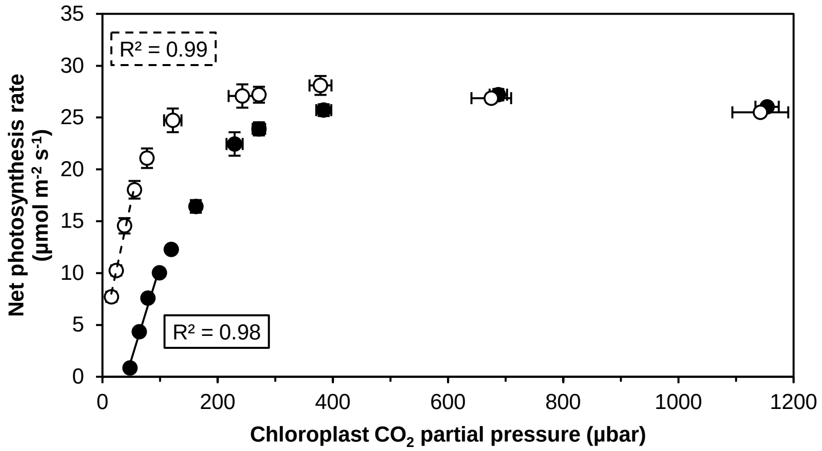

Fig. S1. Data used for determination of the parameters V_{Cmax}, J_{max}, *TPU* and Γ^*. A/C_c relationships in 21% (closed circles) and 2% oxygen (open circles). Leaves were exposed to 11 different CO_2 partial pressures between 50 and 1500 µbar. Data were logged every 5 seconds, and averages of 10 values at each C_a step, after steady-state *A* had visibly been reached, were used. Other cuvette conditions were: 1000 µmol m^{-2} s^{-1} PAR, 0.8 kPa $VPD_{leaf\text{-}air}$ and 23 °C T_{leaf}. Parameters V_{Cmax}, J_{max} and *TPU* were estimated using the curve-fitting procedure by Sharkey *et al*. (2007). Γ^* was calculated using the slopes of the regression lines depicted in the figure, after Yin *et al*. (2009). Average ± SEM (n = 3-5).

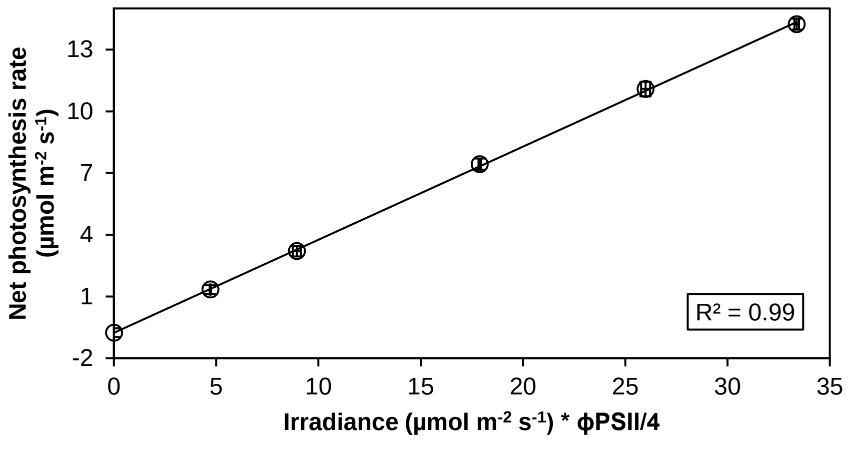

Fig. S2. Data used for the determination of day respiration (R_d). Relationship between net photosynthesis rates and irradiance * Φ_{PSII} * 0.25, as in Yin et al. (2009), measured in 2% O_2. Leaves were adapted to 200 µmol m^{-2} s^{-1}, until *A* and g_s were stable. Then, leaves were exposed to a range of PAR values between 0 and 200 µmol m^{-2} s^{-1}. Data were logged every 5 seconds, and averages of 10 values at each irradiance step, after steady-state *A* had visibly been reached, were used. Other cuvette conditions were: 400 µbar CO_2 partial pressure, 0.8 kPa $VPD_{leaf\text{-}air}$ and 22 °C T_{leaf}. The intercept with the y-axis was assumed to equal R_d (Yin *et al.*, 2009). Average ± SEM (n = 4).

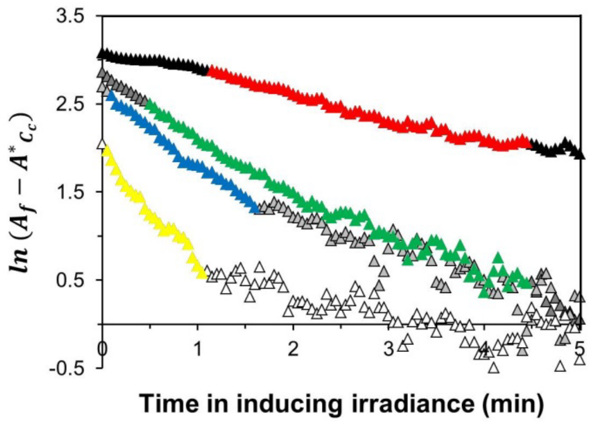

Fig. S3. Examples of determination of the apparent time constant of Rubisco activation (τ_R), in four induction curves (at 400 µbar CO_2), as affected by background irradiance. Data were calculated as the natural logarithm (ln) of the difference of steady-state *A* in inducing irradiance (A_f) and transient *A* after a stepwise increase in irradiance, corrected for changes in chloroplast CO_2 partial pressure ($A^*_{C_c}$). Black-and-white symbols show the complete range of data until 5 minutes after increasing irradiance to 1000 µmol m^{-2} s^{-1}, color symbols show the range chosen for a linear correlation between $\ln(A_f - A^*_{C_i})$ and time. Black and red symbols: 0→1000 µmol m^{-2} s^{-1}, dark grey and green symbols: 50→1000 µmol m^{-2} s^{-1}, light grey and blue symbols: 100→1000 µmol m^{-2} s^{-1}, white and yellow symbols: 200→1000 µmol m^{-2} s^{-1}.

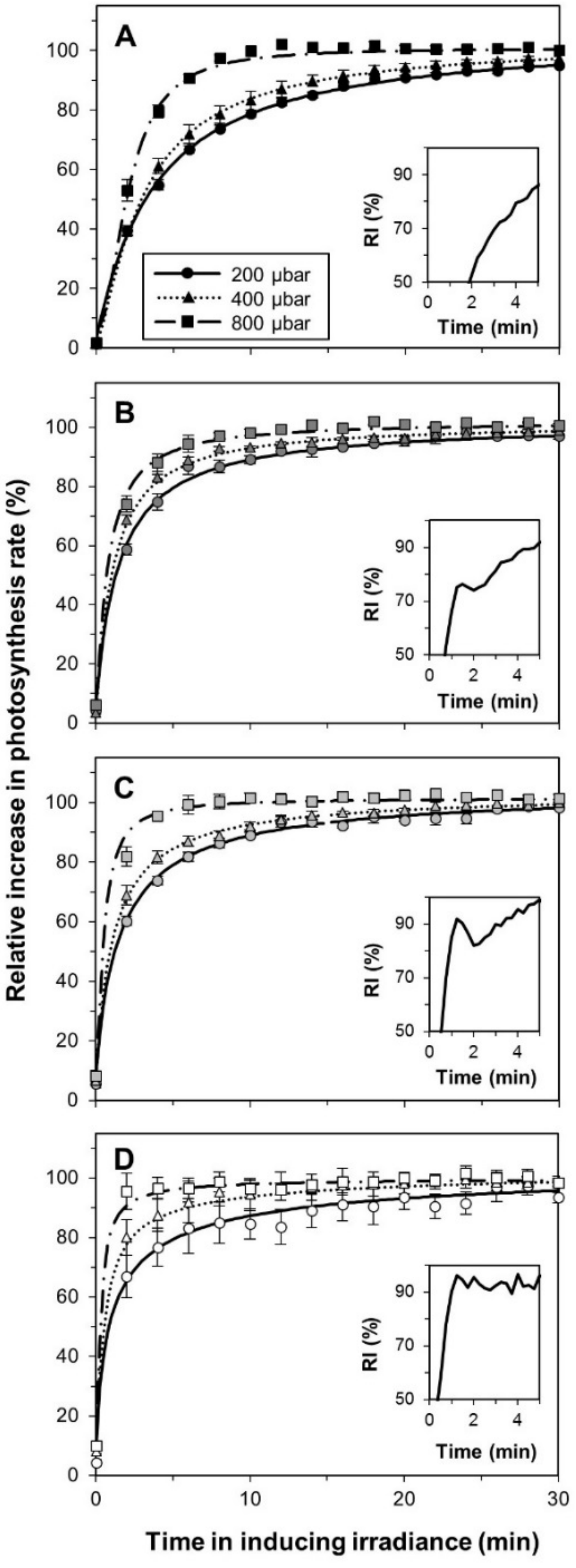

Fig. S4. Relative increases in A after a stepwise increase in irradiance at three CO_2 partial pressures. Background irradiance was 0 (A), 50 (B), 100 (C) or 200 µmol m^{-2} s^{-1} (D); inducing irradiance was 1000 µmol m^{-2} s^{-1}. Lines denote sigmoidal fits (Table S4), symbols denote average ± SEM, n = 3-5. Insets: initial relative increase in A at 800 µbar, to emphasize transient decreases at 50 and 100 µmol m^{-2} s^{-1} background irradiance. The transient decreases depicted in the insets are most likely caused by a transient mismatch between rates of Calvin cycle turnover, sugar synthesis and availability of free phosphate in the chloroplast (Prinsley & Leegood, 1986; Stitt & Grosse, 1988; Stitt & Quick, 1989), due to relatively slow activation of the enzyme sucrose phosphate synthase, which in tomato is light-regulated (Worrell *et al.*, 1991; Galtier *et al.*, 1993). The transient decrease at 800 µbar had negligible effects (at most 3% decrease of integrated *A* in the first 4 minutes after irradiance increases) and was therefore not taken into account in sigmoidal fits.

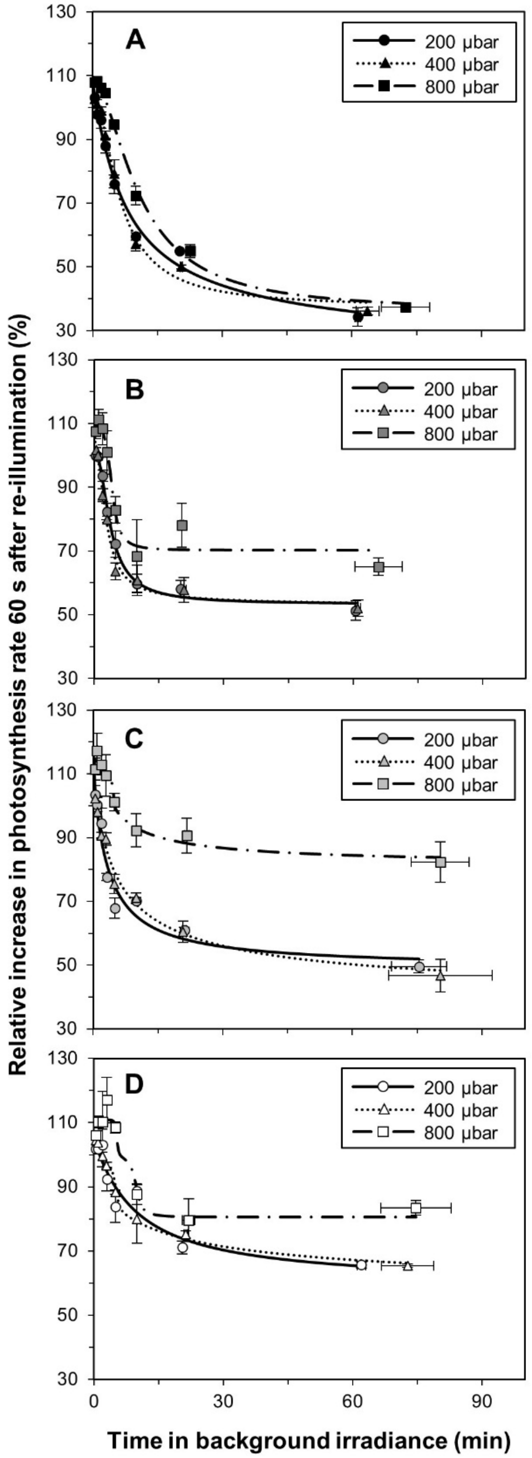

Fig. S5. Loss of photosynthetic induction at different background irradiance levels after stepwise decreases in irradiance at three CO_2 partial pressures. Background irradiance was 0 (A), 50 (B), 100 (C) or 200 µmol m^{-2} s^{-1} (D); inducing irradiance was 1000 µmol m^{-2} s^{-1}. Loss of photosynthetic induction is depicted as the relative increase in *A* 60 s after re-illumination (RI_{60}). Lines denote sigmoidal fits (Table S4), symbols denote average ± SEM, n = 3-4

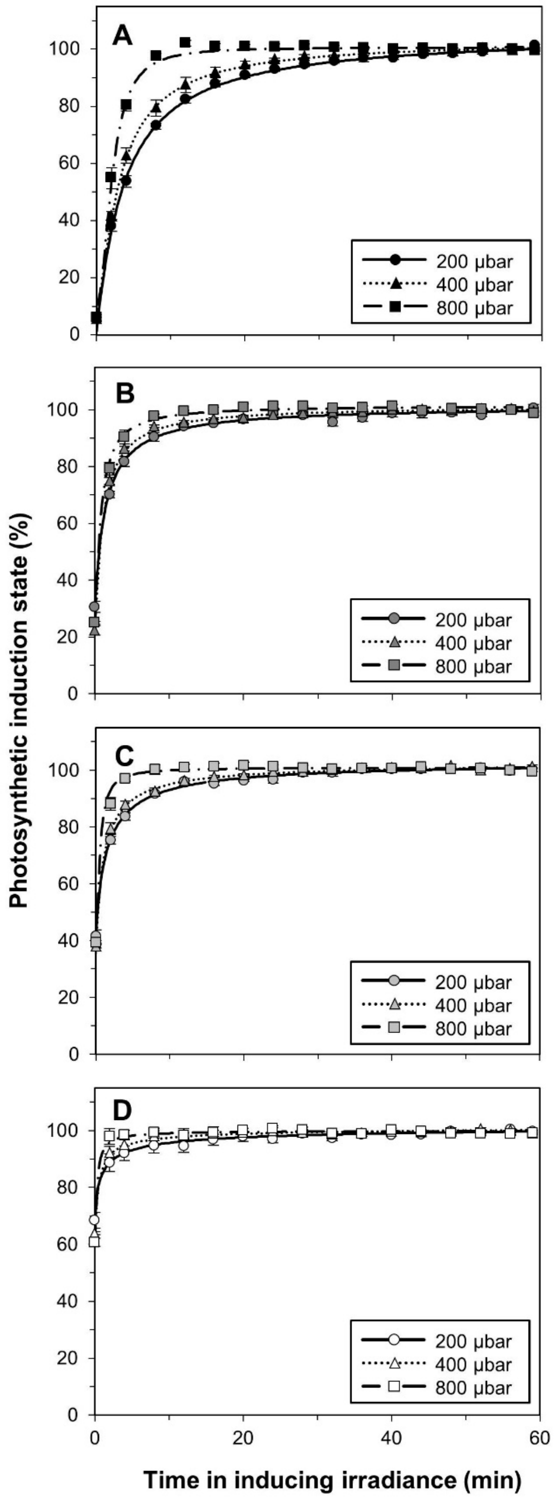

Fig. S6. Photosynthetic induction state (PI) during photosynthetic induction after a stepwise increase in irradiance at three CO_2 partial pressures. Background irradiance was 0 (A), 50 (B), 100 (C) or 200 µmol m^{-2} s^{-1} (D); inducing irradiance was 1000 µmol m^{-2} s^{-1}. Lines denote sigmoidal fits, symbols average ± SEM, n = 3-5

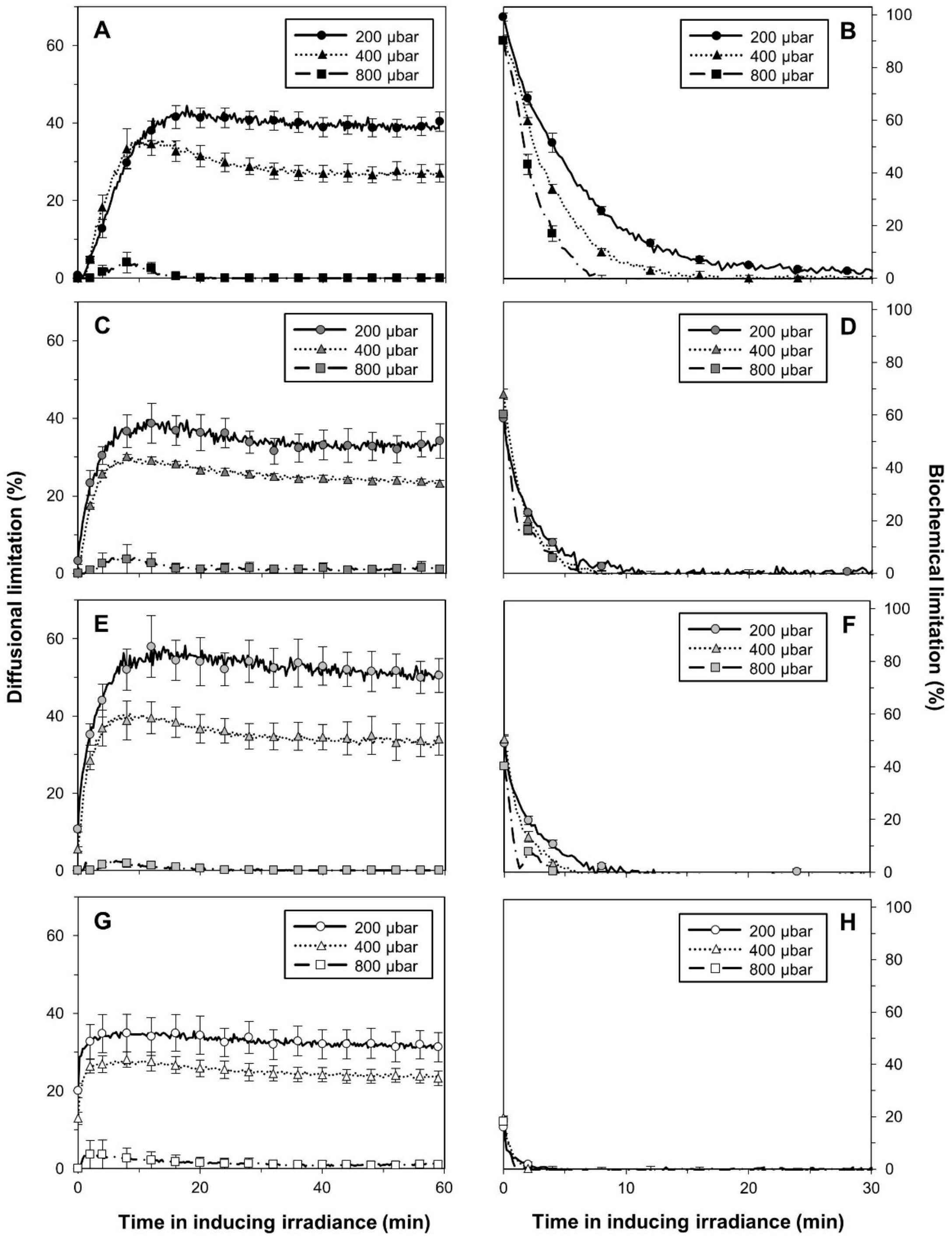

Fig. S7. Changes in diffusional (left panel) and biochemical limitation (right panel) after a stepwise increase in irradiance at three CO_2 partial pressures. Background irradiance was 0 (A, B), 50 (C, D), 100 (E, F) or 200 µmol m^{-2} s^{-1} (G, H); inducing irradiance was 1000 µmol m^{-2} s^{-1}. Lines and symbols denote average, error bars ± SEM, n = 3-5

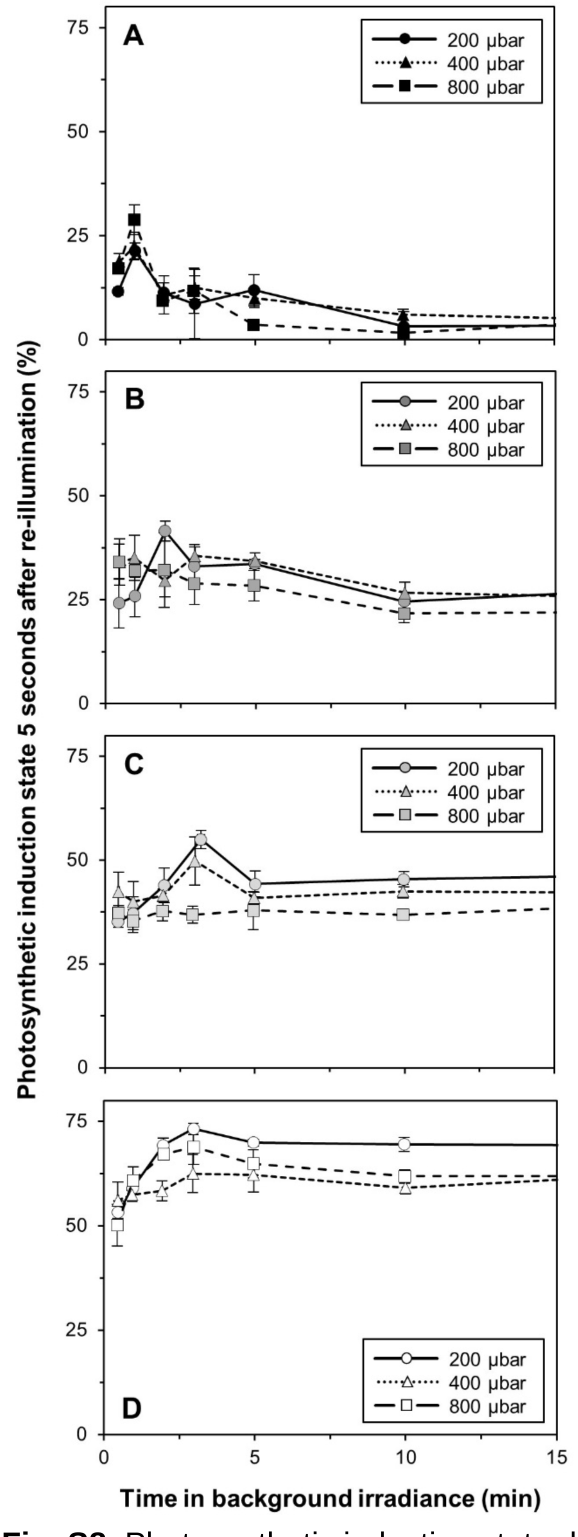

Fig. S8. Photosynthetic induction state during loss of photosynthetic induction in the first 15 minutes after a stepwise decrease in irradiance at three CO_2 partial pressures, shown as photosynthetic induction state 5 seconds after re-illumination. Background irradiance was 0 (A), 50 (B), 100 (C) or 200 µmol m^{-2} s^{-1} (D); inducing irradiance was 1000 µmol m^{-2} s^{-1}. Symbols denote average ± SEM, n = 3-4

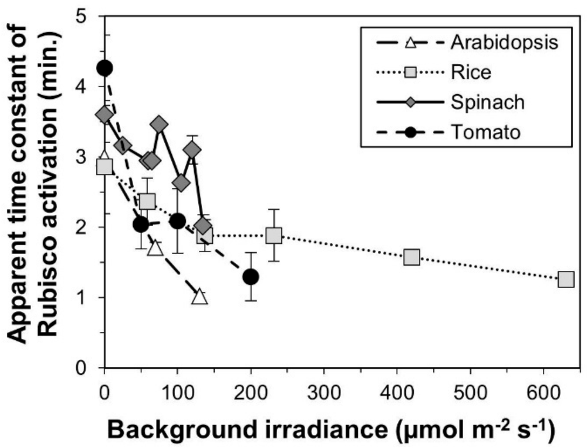

Fig. S9. Comparison of the relationship between τ_R and background irradiance in several species. Data were compiled from the following studies: Kaiser et al. (2016; *A. thaliana*); Fukayama et al. (1998; *Oryza sativa*); Jackson et al. (1991; *Spinacia oleracea*) and the current study (*Solanum lycopersicum*, same as Fig. 6B). Error bars were added as supplied in the original studies. In Jackson et al. (1991), values for single replicates were shown; for data points from the same irradiance, average ± SE was calculated

References

Bernacchi CJ, Singsaas EL, Pimentel C, Portis AR, Long SP. 2001. Improved temperature response functions for models of Rubisco-limited photosynthesis. Plant, Cell and Environment **24**, 253–259.

Farquhar GD, von Caemmerer S, Berry JA. 1980. A biochemical model of photosynthetic CO_2 assimilation in leaves of C_3 species. Planta **149**, 78–90.

Fukayama H, Uchida N, Azuma T, Yasuda T. 1998. Light-dependent activation of CO_2 assimilation and the ratio of Rubisco activase to Rubisco during leaf aging of rice (*Oryza sativa*). Physiologia Plantarum **104**, 541–548.

Galtier N, Foyer CH, Huber J, Voelker TA, Huber SC. 1993. Effects of elevated sucrose-phosphate synthase activity on photosynthesis, assimilate partitioning, and growth in tomato (*Lycopersicon esculentum* var UC82B). Plant Physiology **101**, 535–543.

Harley PC, Loreto F, Di Marco G, Sharkey TD. 1992. Theoretical considerations when estimating the mesophyll conductance to CO_2 flux by analysis of the response of photosynthesis to CO_2. Plant Physiology **98**, 1429–1436.

Jackson RB, Woodrow IE, Mott KA. 1991. Nonsteady-state photosynthesis following an increase in photon flux density (PFD): Effects of magnitude and duration of initial PFD. Plant Physiology **95**, 498–503.

Kaiser E, Kromdijk J, Harbinson J, Heuvelink E, Marcelis LFM. 2017. Photosynthetic induction and its diffusional, carboxylation and electron transport processes as affected by CO_2 partial pressure, temperature, air humidity and blue irradiance. Annals of Botany **119**, 191–205.

Kaiser E, Morales A, Harbinson J, Heuvelink E, Prinzenberg AE, Marcelis LFM. 2016. Metabolic and diffusional limitations of photosynthesis in fluctuating irradiance in Arabidopsis thaliana. Scientific Reports doi: 10.1038/srep31252

Pons TL, Welschen RAM. 2002. Overestimation of respiration rates in commercially available clamp-on leaf chambers. Complications with measurement of net photosynthesis. Plant, Cell and Environment **25**, 1367–1372.

Prinsley RT, Leegood RC. 1986. Factors affecting photosynthetic induction in spinach leaves. Biochimica et Biophysica Acta **849**, 244–253.

Sharkey TD. 1985. O_2-insensitive photosynthesis in C_3 plants. Its occurrence and a possible explanation. Plant Physiology **78**, 71–75.

Sharkey TD, Bernacchi CJ, Farquhar GD, Singsaas EL. 2007. Fitting photosynthetic carbon dioxide response curves for C_3 leaves. Plant, Cell and Environment **30**, 1035–1040.

Stitt M, Grosse H. 1988. Interactions between sucrose synthesis and CO_2 fixation I. Secondary kinetics during photosynthetic induction are related to a delayed activation of sucrose synthesis. Journal of Plant Physiology **133**, 129–137.

Stitt M, Quick WP. 1989. Photosynthetic carbon partitioning: its regulation and posibilities for manipulation. Physiologia Plantarum **77**, 633–641.

Urban O, Košvancová M, Marek M V., Lichtenthaler HK. 2007. Induction of photosynthesis and importance of limitations during the induction phase in sun and shade leaves of five ecologically contrasting tree species from the temperate zone. Tree Physiology, 1207–1215.

Woodrow IE, Mott KA. 1989. Rate limitation of non-steady-state photosynthesis by ribulose-1,5-bisphosphate carboxylase in spinach. Australian Journal of Plant Physiology **16**, 487–500.

Worrell AC, Bruneau JM, Summerfelt K, Boersig M, Voelker TA. 1991. Expression of a maize sucrose phosphate synthase in tomato alters leaf carbohydrate partitioning. The Plant Cell **3**, 1121–1130.

Yin X, Struik PC, Romero P, Harbinson J, Evers JB, Van Der Putten PEL, Vos J. 2009. Using combined measurements of gas exchange and chlorophyll fluorescence to estimate parameters of a biochemical C_3 photosynthesis model: a critical appraisal and a new integrated approach applied to leaves in a wheat (*Triticum aestivum*) canopy. Plant, Cell & Environment **32**, 448–464.
